# Phenyl *N*-(2-methyl­phen­yl)carbamate

**DOI:** 10.1107/S1600536809022788

**Published:** 2009-06-20

**Authors:** Durre Shahwar, M. Nawaz Tahir, Naeem Ahmad, Asma Yasmeen, Saif Ullah

**Affiliations:** a Department of Chemistry, Government College University, Lahore, Pakistan; b Department of Physics, University of Sargodha, Sargodha, Pakistan

## Abstract

In the title compound, C_14_H_13_NO_2_, the aromatic rings attached to the O and N atoms make dihedral angles of 62.65 (9) and 38.28 (11)°, respectively, with the central carbamate group. The benzene rings are oriented at a dihedral angle of 39.22 (10)°. In the crystal, a very weak C—H⋯π inter­action occurs.

## Related literature

For a related structure, see: Shahwar *et al.* (2009 ▶).
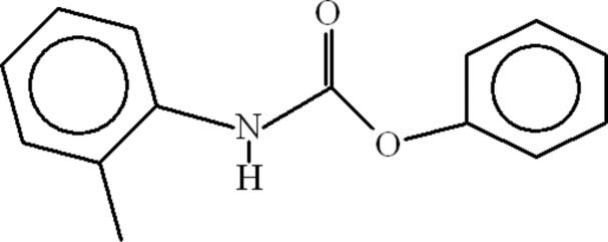


## Experimental

### 

#### Crystal data

C_14_H_13_NO_2_
*M* *_r_* = 227.25
Orthorhombic, 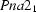

*a* = 10.5736 (9) Å
*b* = 18.5414 (14) Å
*c* = 5.9681 (4) Å
*V* = 1170.04 (15) Å^3^
*Z* = 4
Mo *K*α radiation
μ = 0.09 mm^−1^
*T* = 296 K
0.25 × 0.14 × 0.14 mm

#### Data collection

Bruker Kappa APEXII CCD diffractometer
Absorption correction: multi-scan (*SADABS*; Bruker, 2005 ▶) *T*_min_ = 0.984, *T*_max_ = 0.989
6929 measured reflections
1585 independent reflections
997 reflections with *I* > 2σ(*I*)
*R*_int_ = 0.037

#### Refinement

*R*[*F* ^2^ > 2σ(*F* ^2^)] = 0.039
*wR*(*F* ^2^) = 0.083
*S* = 1.01
1585 reflections
158 parameters
1 restraint
H atoms treated by a mixture of independent and constrained refinement
Δρ_max_ = 0.13 e Å^−3^
Δρ_min_ = −0.15 e Å^−3^

### 

Data collection: *APEX2* (Bruker, 2007 ▶); cell refinement: *SAINT* (Bruker, 2007 ▶); data reduction: *SAINT*; program(s) used to solve structure: *SHELXS97* (Sheldrick, 2008 ▶); program(s) used to refine structure: *SHELXL97* (Sheldrick, 2008 ▶); molecular graphics: *ORTEP-3* (Farrugia, 1997 ▶) and *PLATON* (Spek, 2009 ▶); software used to prepare material for publication: *WinGX* (Farrugia, 1999 ▶) and *PLATON*.

## Supplementary Material

Crystal structure: contains datablocks global, I. DOI: 10.1107/S1600536809022788/hb5008sup1.cif

Structure factors: contains datablocks I. DOI: 10.1107/S1600536809022788/hb5008Isup2.hkl

Additional supplementary materials:  crystallographic information; 3D view; checkCIF report

## Figures and Tables

**Table 1:** Hydrogen-bond geometry (Å, °)

*D*—H⋯*A*	*D*—H	H⋯*A*	*D*⋯*A*	*D*—H⋯*A*
C5—H5⋯CgB^i^	0.93	2.95	3.714 (3)	140

Symmetry code: (i) 
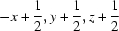
. *CgB* is the centroid of benzene ring (C8–C13).

**Fig. 1.**
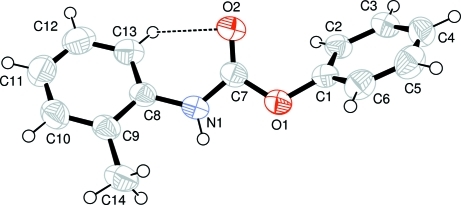
View of (I) with displacement ellipsoids drawn at the 50% probability level. H-atoms are shown by small spheres of arbitrary radius.

## supplementary crystallographic information

### Comment

We have recently published the crystal structure of (II), phenyl *N*-phenylcarbamate (Shahwar *et al.*, 2009), which differs from the title compound, (I), due to an attachement of CH_3_ at *ortho*-position of benzene ring attached with N-atom.

In (I), the benzene rings A (C1—C6) and B (C8—C13) are of course planar. The central portion containing carbamate group C (C7/O1/O2/N1) is also planar. The benzene rings A & B are oriented at a dihedral angle of 39.22 (10)°. The dihedral angles between A/C and B/C have values of of 62.65 (9)° and 38.28 (11)°, respectively. The H-atom attached with N-atom does not form any intera or inter-molecular H-bonding due to the attachement of methyl group. There exists a weak C–H···π interaction (Table 1).

### Experimental

A solution of *o*-toluidine (1.08 ml, 0.01 mol) in dichloromethane (20 ml) was prepared. Phenylchloroformate (1.26 ml, 0.01 mol) was added drop-wise to the magnetically stirring solution. The mixture turned to suspension after one hour.  To get complete product, n-hexane (30 ml) was added and the precipitate were obtained. The precipitate were filtered out and recrystalized from ethylacetate and methanol (9:1) to yield colourless blocks of (I).

### Refinement

In the absence of significant anomalous scattering effects, Friedel pairs were merged before refinement.

The coordinates of the N-bound H atom were refined. The C-bound H atoms were positioned geometrically (C—H = 0.93–0.96 Å) and refined as riding with U_iso_(H) = 1.2U_eq_(C, N) or 1.5U_eq_(methyl C).

### Crystal data

**Table:**

C_14_H_13_NO_2_	*F*(000) = 480
*M**_r_* = 227.25	*D*_x_ = 1.290 Mg m^−^^3^
Orthorhombic, *P**n**a*2_1_	Mo *K*α radiation, λ = 0.71073 Å
Hall symbol: P 2c -2n	Cell parameters from 2241 reflections
*a* = 10.5736 (9) Å	θ = 3.0–28.6°
*b* = 18.5414 (14) Å	µ = 0.09 mm^−^^1^
*c* = 5.9681 (4) Å	*T* = 296 K
*V* = 1170.04 (15) Å^3^	Block, colourless
*Z* = 4	0.25 × 0.14 × 0.14 mm

### Data collection

**Table:**

Bruker Kappa APEXII CCD diffractometer	1585 independent reflections
Radiation source: fine-focus sealed tube	997 reflections with *I* > 2σ(*I*)
graphite	*R*_int_ = 0.037
Detector resolution: 7.40 pixels mm^-1^	θ_max_ = 28.3°, θ_min_ = 2.9°
ω scans	*h* = −14→13
Absorption correction: multi-scan (*SADABS*; Bruker, 2005)	*k* = −17→24
*T*_min_ = 0.984, *T*_max_ = 0.989	*l* = −7→7
6929 measured reflections	

### Refinement

**Table:**

Refinement on *F*^2^	Primary atom site location: structure-invariant direct methods
Least-squares matrix: full	Secondary atom site location: difference Fourier map
*R*[*F*^2^ > 2σ(*F*^2^)] = 0.039	Hydrogen site location: inferred from neighbouring sites
*wR*(*F*^2^) = 0.083	H atoms treated by a mixture of independent and constrained refinement
*S* = 1.01	*w* = 1/[σ^2^(*F*_o_^2^) + (0.0377*P*)^2^] where *P* = (*F*_o_^2^ + 2*F*_c_^2^)/3
1585 reflections	(Δ/σ)_max_ < 0.001
158 parameters	Δρ_max_ = 0.13 e Å^−^^3^
1 restraint	Δρ_min_ = −0.15 e Å^−^^3^

### Special details

**Table:**

Geometry. Bond distances, angles *etc*. have been calculated using the rounded fractional coordinates. All su's are estimated from the variances of the (full) variance-covariance matrix. The cell e.s.d.'s are taken into account in the estimation of distances, angles and torsion angles
Refinement. Refinement of *F*^2^ against ALL reflections. The weighted *R*-factor *wR* and goodness of fit *S* are based on *F*^2^, conventional *R*-factors *R* are based on *F*, with *F* set to zero for negative *F*^2^. The threshold expression of *F*^2^ > σ(*F*^2^) is used only for calculating *R*-factors(gt) *etc*. and is not relevant to the choice of reflections for refinement. *R*-factors based on *F*^2^ are statistically about twice as large as those based on *F*, and *R*- factors based on ALL data will be even larger.

### Fractional atomic coordinates and isotropic or equivalent isotropic displacement parameters (Å^2^)

**Table:**

	*x*	*y*	*z*	*U*_iso_*/*U*_eq_	
O1	0.27882 (15)	0.33744 (9)	0.6965 (3)	0.0594 (6)	
O2	0.48038 (14)	0.30458 (9)	0.6133 (3)	0.0585 (6)	
N1	0.30983 (19)	0.24161 (11)	0.4867 (3)	0.0547 (7)	
C1	0.31727 (19)	0.39161 (13)	0.8463 (4)	0.0459 (8)	
C2	0.3813 (2)	0.37450 (12)	1.0394 (4)	0.0492 (8)	
C3	0.4089 (2)	0.42831 (13)	1.1897 (4)	0.0550 (9)	
C4	0.3718 (2)	0.49827 (14)	1.1487 (5)	0.0610 (9)	
C5	0.3068 (2)	0.51391 (15)	0.9561 (4)	0.0657 (10)	
C6	0.2797 (2)	0.46077 (14)	0.8027 (4)	0.0580 (9)	
C7	0.3691 (2)	0.29463 (12)	0.6005 (4)	0.0472 (8)	
C8	0.3694 (2)	0.19088 (12)	0.3442 (4)	0.0479 (7)	
C9	0.3097 (2)	0.17293 (13)	0.1431 (4)	0.0508 (8)	
C10	0.3682 (3)	0.12288 (15)	0.0089 (4)	0.0683 (10)	
C11	0.4814 (3)	0.09134 (15)	0.0662 (6)	0.0783 (12)	
C12	0.5378 (3)	0.10948 (16)	0.2643 (6)	0.0746 (11)	
C13	0.4824 (2)	0.15870 (14)	0.4054 (5)	0.0599 (9)	
C14	0.1842 (2)	0.20522 (15)	0.0812 (5)	0.0670 (10)	
H1	0.233 (2)	0.2453 (13)	0.486 (5)	0.0657*	
H2	0.40570	0.32718	1.06787	0.0590*	
H3	0.45290	0.41743	1.32027	0.0660*	
H4	0.39062	0.53459	1.25089	0.0732*	
H5	0.28080	0.56101	0.92870	0.0787*	
H6	0.23641	0.47170	0.67139	0.0696*	
H10	0.33000	0.10987	−0.12545	0.0820*	
H11	0.51913	0.05797	−0.02901	0.0938*	
H12	0.61432	0.08830	0.30394	0.0895*	
H13	0.52051	0.17034	0.54111	0.0718*	
H14A	0.12302	0.19369	0.19447	0.1006*	
H14B	0.15695	0.18596	−0.06011	0.1006*	
H14C	0.19249	0.25665	0.06956	0.1006*	

### Atomic displacement parameters (Å^2^)

**Table:**

	*U*^11^	*U*^22^	*U*^33^	*U*^12^	*U*^13^	*U*^23^
O1	0.0456 (10)	0.0665 (11)	0.0662 (11)	0.0037 (8)	−0.0135 (9)	−0.0153 (10)
O2	0.0448 (10)	0.0669 (11)	0.0638 (10)	−0.0087 (8)	−0.0047 (9)	−0.0083 (9)
N1	0.0419 (11)	0.0632 (13)	0.0591 (12)	−0.0031 (11)	−0.0084 (12)	−0.0090 (11)
C1	0.0369 (12)	0.0511 (14)	0.0497 (14)	−0.0005 (10)	−0.0010 (11)	0.0007 (12)
C2	0.0439 (13)	0.0417 (13)	0.0620 (15)	−0.0029 (11)	−0.0062 (11)	0.0052 (12)
C3	0.0509 (15)	0.0604 (16)	0.0538 (14)	−0.0063 (12)	−0.0084 (12)	−0.0008 (14)
C4	0.0625 (15)	0.0521 (15)	0.0683 (18)	−0.0070 (12)	0.0069 (14)	−0.0096 (14)
C5	0.0692 (19)	0.0490 (15)	0.079 (2)	0.0116 (13)	0.0064 (15)	0.0095 (15)
C6	0.0577 (16)	0.0621 (17)	0.0541 (16)	0.0100 (13)	−0.0013 (13)	0.0092 (14)
C7	0.0484 (14)	0.0513 (14)	0.0418 (11)	−0.0019 (12)	−0.0080 (12)	0.0035 (11)
C8	0.0445 (13)	0.0489 (13)	0.0503 (12)	−0.0100 (12)	−0.0001 (12)	0.0023 (12)
C9	0.0479 (14)	0.0562 (14)	0.0484 (14)	−0.0180 (11)	0.0020 (11)	0.0013 (13)
C10	0.0679 (19)	0.0809 (19)	0.0562 (16)	−0.0237 (16)	0.0076 (15)	−0.0113 (15)
C11	0.070 (2)	0.072 (2)	0.093 (2)	−0.0072 (16)	0.0194 (18)	−0.0196 (17)
C12	0.0536 (17)	0.0682 (19)	0.102 (2)	0.0007 (15)	0.0057 (17)	−0.0014 (18)
C13	0.0509 (16)	0.0598 (17)	0.0689 (16)	−0.0023 (12)	−0.0067 (13)	0.0042 (14)
C14	0.0568 (15)	0.084 (2)	0.0601 (14)	−0.0133 (14)	−0.0147 (13)	0.0003 (15)

### Geometric parameters (Å, °)

**Table:**

O1—C1	1.405 (3)	C10—C11	1.375 (4)
O1—C7	1.367 (3)	C11—C12	1.366 (5)
O2—C7	1.194 (3)	C12—C13	1.373 (4)
N1—C7	1.349 (3)	C2—H2	0.9300
N1—C8	1.416 (3)	C3—H3	0.9300
N1—H1	0.82 (2)	C4—H4	0.9300
C1—C6	1.367 (3)	C5—H5	0.9300
C1—C2	1.374 (3)	C6—H6	0.9300
C2—C3	1.373 (3)	C10—H10	0.9300
C3—C4	1.377 (4)	C11—H11	0.9300
C4—C5	1.370 (4)	C12—H12	0.9300
C5—C6	1.375 (4)	C13—H13	0.9300
C8—C13	1.385 (3)	C14—H14A	0.9600
C8—C9	1.396 (3)	C14—H14B	0.9600
C9—C10	1.373 (4)	C14—H14C	0.9600
C9—C14	1.502 (3)		
			
C1—O1—C7	118.67 (17)	C1—C2—H2	121.00
C7—N1—C8	125.43 (19)	C3—C2—H2	120.00
C8—N1—H1	119.7 (19)	C2—C3—H3	120.00
C7—N1—H1	113.9 (18)	C4—C3—H3	120.00
O1—C1—C6	117.7 (2)	C3—C4—H4	120.00
C2—C1—C6	121.3 (2)	C5—C4—H4	120.00
O1—C1—C2	120.8 (2)	C4—C5—H5	120.00
C1—C2—C3	119.0 (2)	C6—C5—H5	120.00
C2—C3—C4	120.5 (2)	C1—C6—H6	121.00
C3—C4—C5	119.4 (2)	C5—C6—H6	120.00
C4—C5—C6	120.8 (2)	C9—C10—H10	119.00
C1—C6—C5	119.0 (2)	C11—C10—H10	119.00
O1—C7—N1	108.04 (18)	C10—C11—H11	120.00
O2—C7—N1	127.1 (2)	C12—C11—H11	120.00
O1—C7—O2	124.9 (2)	C11—C12—H12	120.00
C9—C8—C13	120.9 (2)	C13—C12—H12	120.00
N1—C8—C9	118.26 (19)	C8—C13—H13	120.00
N1—C8—C13	120.8 (2)	C12—C13—H13	120.00
C10—C9—C14	121.6 (2)	C9—C14—H14A	109.00
C8—C9—C10	117.3 (2)	C9—C14—H14B	109.00
C8—C9—C14	121.1 (2)	C9—C14—H14C	109.00
C9—C10—C11	122.3 (3)	H14A—C14—H14B	109.00
C10—C11—C12	119.4 (3)	H14A—C14—H14C	109.00
C11—C12—C13	120.5 (3)	H14B—C14—H14C	109.00
C8—C13—C12	119.5 (3)		
			
C7—O1—C1—C2	−60.4 (3)	C3—C4—C5—C6	−0.8 (3)
C7—O1—C1—C6	124.9 (2)	C4—C5—C6—C1	0.8 (3)
C1—O1—C7—O2	−9.1 (3)	N1—C8—C9—C10	−179.1 (2)
C1—O1—C7—N1	172.51 (19)	N1—C8—C9—C14	−1.3 (3)
C8—N1—C7—O1	172.7 (2)	C13—C8—C9—C10	−0.6 (4)
C8—N1—C7—O2	−5.7 (4)	C13—C8—C9—C14	177.2 (2)
C7—N1—C8—C9	−139.0 (2)	N1—C8—C13—C12	179.8 (2)
C7—N1—C8—C13	42.5 (3)	C9—C8—C13—C12	1.3 (4)
O1—C1—C2—C3	−175.14 (19)	C8—C9—C10—C11	−0.4 (4)
C6—C1—C2—C3	−0.7 (3)	C14—C9—C10—C11	−178.2 (3)
O1—C1—C6—C5	174.57 (19)	C9—C10—C11—C12	0.7 (5)
C2—C1—C6—C5	−0.1 (3)	C10—C11—C12—C13	0.0 (5)
C1—C2—C3—C4	0.7 (3)	C11—C12—C13—C8	−1.0 (4)
C2—C3—C4—C5	0.0 (3)		

### Hydrogen-bond geometry (Å, °)

**Table:**

*D*—H···*A*	*D*—H	H···*A*	*D*···*A*	*D*—H···*A*
C5—H5···CgB^i^	0.93	2.95	3.714 (3)	140

Symmetry codes: (i) −*x*+1/2, *y*+1/2, *z*+1/2.
